# Successful Endoscopic Treatment of Bouveret Syndrome in a Patient with Choledochoduodenal Fistula Complicating Duodenal Ulcer

**DOI:** 10.1155/2017/6918905

**Published:** 2017-06-19

**Authors:** Syed Hasan, Zubair Khan, Umar Darr, Toseef Javaid, Nauman Siddiqui, Jamal Saleh, Abdallah Kobeissy, Ali Nawras

**Affiliations:** ^1^Department of Internal Medicine, University of Toledo Medical Center, Toledo, OH, USA; ^2^Division of Gastroenterology, University of Toledo, Toledo, OH, USA

## Abstract

**Introduction:**

Cholecystoduodenal fistulas represent the most common type of bilioenteric fistulas while choledochoduodenal fistulas account for only 1–25% of cases. Bilioenteric fistula cases are associated with cholelithiasis and are rarely associated with duodenal peptic ulcers. Here we present the first case of Bouveret syndrome secondary to choledochoduodenal fistula complicating peptic duodenal ulcer managed successfully via endoscopic mechanical lithotripsy.

**Case:**

86-year-old male with a medical history significant for coronary artery disease and stage 3 colorectal cancer status after resection and chemoradiation presented with intractable sharp abdominal pain worse postprandially for one week in duration, associated with early satiety, anorexia, and 5 lbs weight loss in one week. CT abdomen showed possible choledochoduodenal fistula and a distended stomach. An esophagogastroduodenoscopy (EGD) was performed revealing a large 2.5–3 cm stone lodged in the duodenal bulb at the base of duodenal ulcer with a fistula opening beneath it. The stone was extracted in 2 pieces via mechanical lithotripsy. Endoscopic ultrasound of the CBD revealed Rigler's triad.

**Conclusion:**

Bouveret syndrome is mostly associated with cholecystoduodenal fistula and has high mortality and morbidity due to underlying comorbid conditions and elderly age. Patients are not always fit for surgical management, and endoscopic management is not always successful.

## 1. Introduction

Cholecystoduodenal fistulas represent the most common type of bilioenteric fistulas while choledochoduodenal fistulas account for only 1–25% of bilioenteric fistulas cases [1]. Although 75–90% of bilioenteric fistula cases are associated with cholelithiasis [1, 2] only 5-6% of them are associated with duodenal peptic ulcers [2–4].

The passage of a large gallstone through a cholecystoduodenal fistula and the subsequent impaction in the duodenum causing gastric outlet obstruction are a rare occurrence and this is known as Bouveret syndrome. This type of gallstone ileus was first described by the Beaussier in 1770 and again by Leon Bouveret in 1896 [5]. It is more prevalent in the elderly and in females, with a reported median age of 74 years and a female-to-male ratio of 1.9 [6–9]. It is mostly caused by cholecystoduodenal fistula or rarely cholecystogastric fistula.

Because of the older age and significant comorbid conditions in patients presenting with Bouveret syndrome, the mortality (60%) and morbidity (12–33%) are relatively high and necessitate early and quick removal of the stone by the least invasive procedure [5, 10].

## 2. Case Study

**Figure 1:**
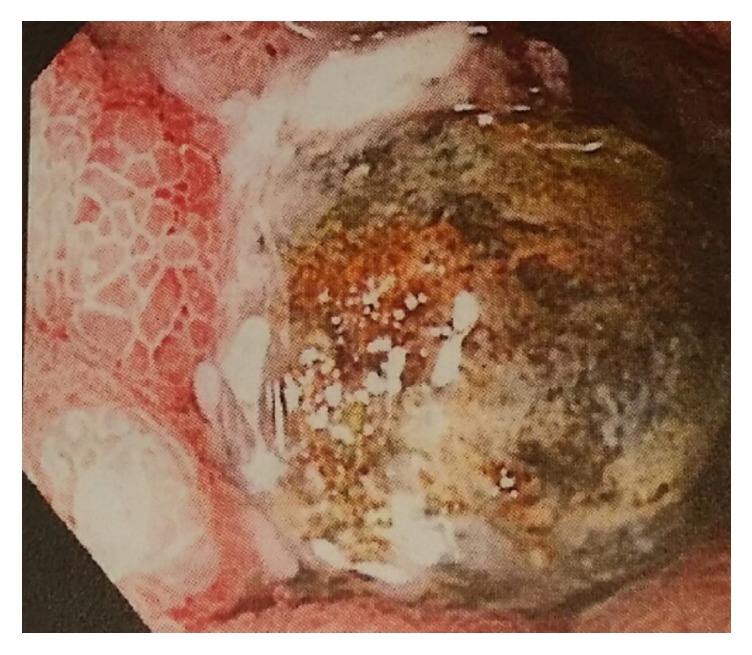
Impacted gallstone in duodenum.

This is an 86-year-old male patient with a past medical history significant for coronary artery disease and stage 3 colorectal cancer status after resection and chemoradiation that presented from an outlying facility for intractable sharp abdominal pain that was worse postprandially, one week in duration, associated with early satiety, anorexia, and 5 lbs weight loss in the last week. Physical exam revealed mild-to-moderate epigastric tenderness and right hypochondrial tenderness. The differential diagnosis before any further work-up was peptic ulcer disease, cholecystitis, or metastasis from colorectal cancer to gastroduodenal region. A CT scan of the abdomen at the outlying facility showed possible choledochoduodenal fistula, distended stomach, and a cyst in the tail of the pancreas. An esophagogastroduodenoscopy (EDG) was performed revealing a large duodenal bulb ulcer with a stone lodged in it (Figure 1).

**Figure 2:**
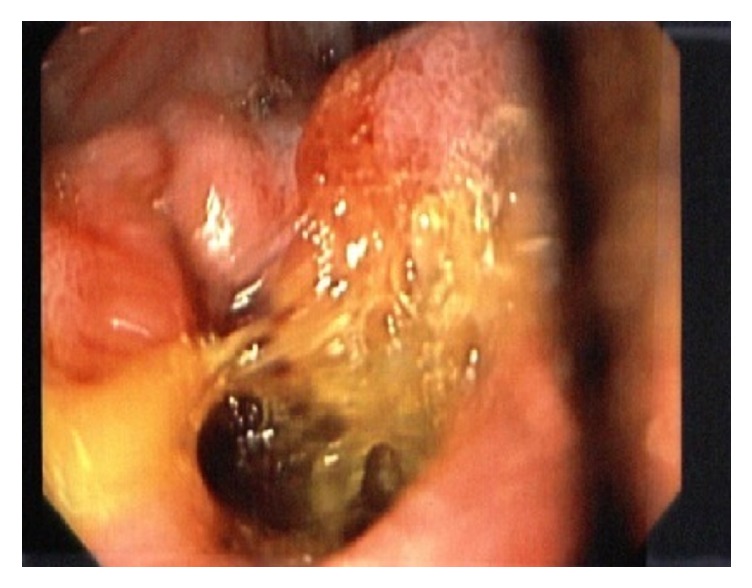
Choledochoduodenal fistula at the base of ulcer.

The ulcer was 1.5 × 0.5 cm and the suspected choledochoduodenal fistula was identified (Figure 2). The stone was secured with a Roth net, and extraction was attempted.

**Figure 3:**
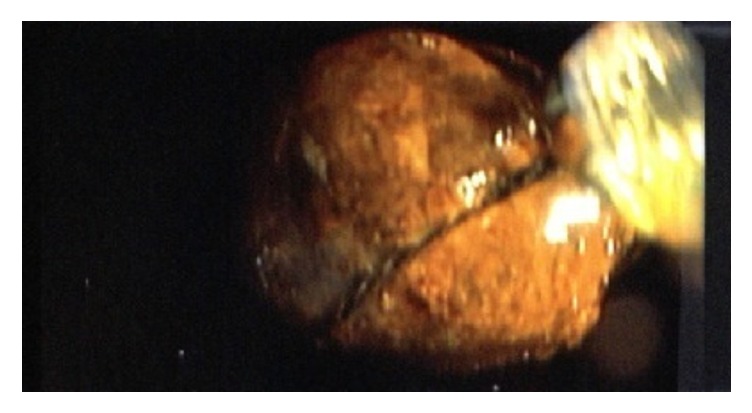
Stone in lithotripter.

**Figure 4:**
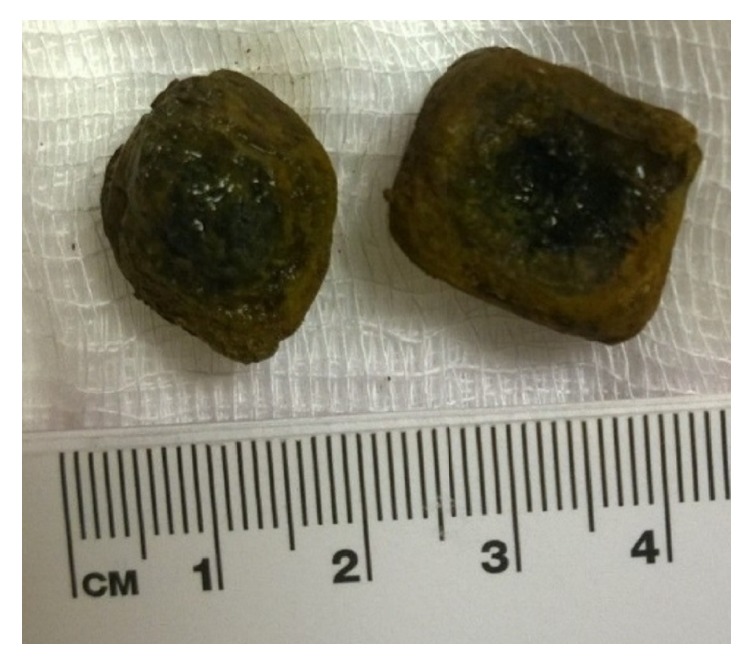
Broken gallstone.

The stone was pulled successfully into the stomach but could not be pulled through the esophagus without a significant risk of traumatizing the esophagus because of the size of the stone. Thus, it was mechanically crushed into 2 pieces using a biliary mechanical lithotripter and extracted (Figure 3). The stone measured 2.5 × 3 cm in diameter (Figure 4).

The duodenum was then reexamined and a duodenal bulb fistula tract orifice was found on the posterior wall at the base of the ulcer. Examination of the common bile duct (CBD) with endoscopic ultrasound (EUS) revealed regions of high echogenicity with prominent shadowing consistent with pneumobilia and no gallstones in the gall bladder. The patient did well after the procedure and his symptoms were completely resolved. He subsequently underwent cholecystectomy and was discharged home on proton pump inhibitors for 8 weeks after one week of hospital stay. He underwent follow-up EGD after 8 weeks at the outside facility that reported resolution of duodenal bulb ulcer and choledochoduodenal fistula. He also underwent HIDA scan and that did not show any biliary-enteric leak.

## 3. Discussion

The creation of a bilioenteric fistula is a very rare complication of cholelithiasis which affects less than 1% of patients [11]. The fistula can occur anywhere in the GI tract with the most common location being cholecystoduodenal (~60%) and cholecystocolic (17%) and cholecystogastric and choledochoduodenal fistulas (5%) [5]. The risk factors for developing bilioenteric fistulas in patients with cholelithiasis include long standing cholelithiasis, repetitive bouts of acute cholecystitis, being a female, age > 60 yrs, and a large calculus (greater than 2 cm) [12].

To the best of our knowledge, this is the first reported case of Bouveret syndrome secondary to choledochoduodenal fistula. All the previous reported cases describe Bouveret syndrome mostly secondary to cholecystoduodenal fistula. Most of the bilioenteric fistulas are associated with cholelithiasis but choledochoduodenal fistulas are unique as they are predominantly attributed to duodenal peptic ulcers (75–80%) and are a rare occurrence [1–3]. The reason for the rarity becomes obvious when one realizes that a duodenal ulcer most typically occurs about 4 cm distal to the pylorus whereas the CBD is about 7 cm distal to the pylorus [13]. Most cases of choledochoduodenal fistulas occur at the posterior wall of the duodenal bulb, and fistulas at the anterior wall of the duodenal bulb are extremely rare [1, 2, 14–16]. Another important fact is that Bouveret syndrome is more prevalent in elderly females but choledochoduodenal fistula is more prevalent in elderly males [15].

For the most part, choledochoduodenal fistulas are incidental findings at upper GI studies and seldom produce any specific symptoms; the symptoms if present are either result of biliary tract obstruction or result of duodenal peptic ulcers [13]. In our case the patient was having impacted gallstone at the duodenal ulcer so he presented with symptoms of Bouveret syndrome. In Bouveret syndrome, findings on presentation are often nonspecific with nausea, distention, and abdominal pain being the most common [17]. And, because of the nonspecific and vague presentation, the diagnosis is often delayed. Radiographic imaging of these patients may reveal a radiolucent gallstone, pyloric, or duodenal obstruction and pneumobilia known as Rigler's triad [10, 17]. Our patient had all three major symptoms and Rigler's triad as identified by Cappell and Davis.

The most frequent sites where stones are found to be impacted are the terminal ilium (50–75%) and proximal ileum and jejunum (20–40%) and rarely the stomach and duodenum [12]. On endoscopy, the gastroduodenal obstruction was noted in nearly all the cases but a review of 128 cases by Cappell and Davis showed the stone itself was only visualized in 69% of cases, while the fistula itself was only visualized in 13% of the cases evaluated [17]. In our patient, the stone was found impacted in the duodenal bulb, obstructing the pyloric outlet. While many options exist for lithotripsy, including electrohydraulic, extracorporeal shockwave, and laser, our patient was treated using mechanical lithotripsy. Despite having various endoscopy techniques, the success rate of these is only 9% [18].

Given the elderly patient population of Bouveret syndrome with significant comorbid conditions, sometimes significant delay in diagnosis requires safe and quick removal of stone to relive the obstruction. The endoscopic removal of the stone is a good option. If the stone is too large for removal through endoscope alone, mechanic, laser, or extracorporeal shockwave lithotripsy may be considered [19]. In the presence of skilled endoscopist and proper facilities, endoscopic retrieval of stone is preferred over invasive procedures like enterolithotomy and cholecystectomy, unless the need arises for it because of other symptoms or in the setting of gallbladder malignancy [19, 20].

In our case beside treating the Bouveret syndrome the other management challenge was duodenal peptic ulcer complicating choledochoduodenal fistulas. Mostly isolated choledochoduodenal fistulas with duodenal peptic ulcers are treated medically with proton pump inhibitors. Surgery must be reserved for patients with poorly controlled or recurrent ulcer symptoms, major ulcer complications, such as perforation, hemorrhage, or obstruction, or exceptional cases with cholangitis or biliary obstruction [16, 21]. As in our case the patient presentation was attributable to Bouveret syndrome that was managed successfully endoscopically, no further intervention was done for choledochoduodenal fistulas beside prescribing proton pump inhibitors for duodenal peptic ulcer.

In conclusion, we report a rare case of choledochoduodenal fistula complicating a duodenal peptic ulcer and the first reported case of Bouveret syndrome secondary to choledochoduodenal fistula which was successfully managed endoscopically. The choledochoduodenal fistulas are rare in this modern era because of the exclusive and universal treatment of peptic ulcer disease with proton pump inhibitors. This case further necessitates the need of early utilization of imaging techniques in the evaluation of elderly patient population presenting with nonspecific gastrointestinal symptoms. Also, based on our experience we would recommend treatment of cases of Bouveret syndrome in an institution with skilled endoscopist and advanced facilities as the majority of patient population is not a candidate for invasive interventions.
